# Child poverty is exacerbated by the current war-induced cost-of-living crisis: need for new European-wide measures

**DOI:** 10.1093/eurpub/ckad021

**Published:** 2023-04-18

**Authors:** Aapo Hiilamo, Tea Lallukka

**Affiliations:** Itla Children’s Foundation, Helsinki, Finland; Department of Public Health, Faculty of Medicine, University of Helsinki, Helsinki, Finland

The massive Russian military attack against Ukraine commencing February 2022 has brought about an enormous amount of suffering in Ukraine, Europe and globally. As one of its many downstream effects, the war, in combination with underlying vulnerabilities, has induced a substantial increase in food and energy prices. Price increases in respect of essential goods have affected all of Europe with the highest increases being seen in Eastern Europe. All population groups have been affected to varying extents, but families with children are of particular concern here because they tend to spend a high share of their income on essential goods.

These price increases alongside stagnated incomes imply that low-income families with children must use their emergency savings, take on debt to meet their needs or reduce their consumption of essential things such as healthier food. Moreover, these low-income households do not generally have any emergency savings to fall back on. Taking on debt for essential consumption may lead to over-indebtedness, harming child development in the long run while simply reducing essential consumption increases the risk of food insecurity, compromises children’s healthy development and could lead to social exclusion and anxiety.^1^

The situation is evolving, but it is now evident that child poverty has increased across Europe. While it is difficult to provide real-time estimates of the exact numbers of children falling into poverty, the best, yet still preliminary estimates, suggest that the child poverty rate, measured as the proportion of children in households with less income than is assumed to be needed for minimum needs, may have increased substantially. The highest increases are estimated to be in Hungary (a 17 percentage point increase) and the lowest in Malta (1 percentage point).^2^ In total, the estimated five percentage point increase in the poverty rate in the EU area would imply that there may now be 4 million more children living in poverty. The public health impact of this increase will be significant.

A systematic review of 54 studies found consistent evidence that low family income in childhood has negative causal effects on a wide range of cognitive, health and social outcomes in respect of children’s development.^3^ Meanwhile positive changes in family income are linked to improvements in parental mental health and living environment, indicating causal mechanisms from child poverty to immediate, intermediate and long-term health outcomes.^3^ Evidence indicates that poverty affects children’s cognitive health, including memory and cognitive functions.^1^

The aim of reducing child poverty has been on the EU agenda for some time. The European child guarantee and the European Pillar of Social Rights Action Plan pledge to reduce the number of children at risk of poverty and social exclusion by 5 million, by 2030. However, the current period of price inflation suggests that this goal will become unattainable if no concrete and ground-breaking policies are adopted in response. Given that all EU countries are affected to varying extents, there is a window of opportunity here for EU-wide measures. One policy innovation worth considering is a proposal for an EU-wide child basic income to be paid to all children residing in the EU area. A modest child basic income of a minimum of 50 Euros per month in countries with currently no such universal child benefits has the potential to reduce child poverty significantly within the EU, particularly in countries with the highest child poverty rates^4^ and thus those that are most affected by the current price increases. While the rollout of an EU-wide child basic income programme would require solidarity between the EU countries, it would be a symbolic investment for the future of the ageing continent and alleviate, to some extent, the unequal burden of price increases. Other potentially important European-level measures could include, for example, stronger investments in early childhood education and care to increase parental employment and family income, increased provision of free school meals to alleviate food insecurity and better monitoring of child poverty development at the EU level with new poverty measures to provide an evidence-base for national poverty reduction programmes. While it remains, as yet, uncertain how to quantify the public health impact of these programmes, contingent evidence indicates that such policies may have the potential to induce large public health benefits.^5^
